# Low visual acuity and asteroid hyalosis

**DOI:** 10.11604/pamj.2014.18.247.4917

**Published:** 2014-07-26

**Authors:** Salim Belhassan, Rajaa Daoudi

**Affiliations:** 1 University of Mohamed V souissi, hôpital des Spécialités, Ophtalology A Department

**Keywords:** Asteroid hyalosis, visual acuity, optic correction

## Image in medicine

A 56 years old man was admitted to the ophthalmology department for low visual acuity since his 45 years old with no amelioration after optic correction. The physical exam found visual acuity at 1/10 on the right eye and 9/10 on the left one, clear cornea, intraocular pressure at 14 mmhg on both eyes, the fundus of the right eye showed multiple yellow mobile vitreous particles that are absent on the left fundus. Asteroid hyalosis is a degenerative condition of the eye involving small white opacities in the vitreous humor. Clinically, these opacities are quite refractile, giving the appearance of stars (or asteroids) shining in the night sky except that ocular asteroids are often quite mobile. Ocular asteroids must be distinguished from the more common typical vitreous floaters, which are usually fibrillar or cellular condensates. The cause of asteroid hyalosis is unknown, but it has been associated with diabetes mellitus, hypertension and hypercholesterolemia. The asteroid bodies are made up of hydroxylapatite, which in turn consists of calcium and phosphates or phospholipids. While asteroid hyalosis does not usually severely affect vision, the floating opacities can be quite annoying, and may interfere significantly with visualization and testing of the retina. The treatment of asteroid hyalosis is usually unnecessary, vitrectomy may occasionally be indicated, for both diagnostic and therapeutic purposes.

**Figure 1:**
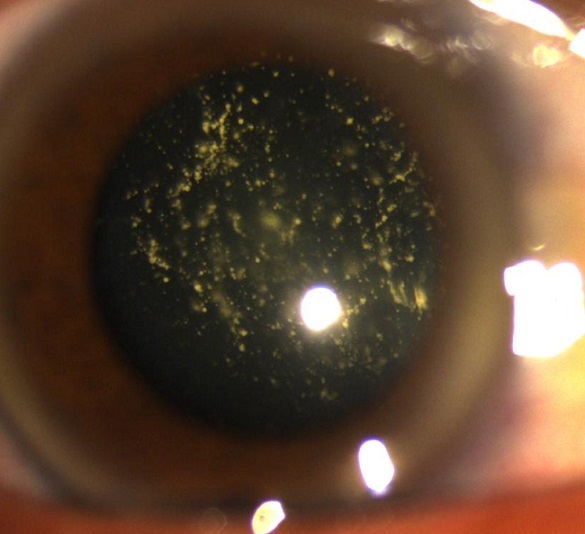
Asteroid hyalosis: multiple yellow mobile vitreous particles

